# Isolation of Bat Sarbecoviruses, Japan

**DOI:** 10.3201/eid2812.220801

**Published:** 2022-12

**Authors:** Shin Murakami, Tomoya Kitamura, Hiromichi Matsugo, Haruhiko Kamiki, Ken Oyabu, Wataru Sekine, Akiko Takenaka-Uema, Yuko Sakai-Tagawa, Yoshihiro Kawaoka, Taisuke Horimoto

**Affiliations:** The University of Tokyo, Tokyo, Japan (S. Murakami, H. Matsugo, H. Kamiki, W. Sekine, A. Takenaka-Uema, Y. Sakai-Tagawa, Y. Kawaoka, T. Horimoto);; National Agriculture and Food Research Organization, Tokyo (T. Kitamura); Isumi County‒City Nature Preservation Association, Isumi-gun, Chiba, Japan (K. Oyabu)

**Keywords:** sarbecovirus, viruses, severe acute respiratory syndrome coronavirus 2, SARS-CoV-2, coronaviruses, respiratory infections, bat, Rhinolophus, betacoronavirus, virus isolation, angiotensin converting enzyme-2, ACE2, SARS-related coronavirus, zoonoses, Japan

## Abstract

Surveillance of bat betacoronaviruses is crucial for understanding their spillover potential. We isolated bat sarbecoviruses from *Rhinolophus cornutus* bats in multiple locations in Japan. These viruses grew efficiently in cells expressing *R. cornutus* angiotensin converting enzyme-2, but not in cells expressing human angiotensin converting enzyme-2, suggesting a narrow host range.

Human betacoronaviruses are divided into 2 pathotypes: endemic viruses, such as human coronavirus OC43 (HCoV-OC43) and HCoV-HKU1, which cause mild respiratory symptoms (*1*), and highly pathogenic viruses comprising severe acute respiratory syndrome coronavirus (SARS-CoV), Middle East respiratory syndrome coronavirus, and SARS-CoV-2, which have caused outbreaks in the past 2 decades (*1*,*2*). Because all these highly pathogenic human betacoronaviruses are considered to have originated from bat-derived viruses (*2*–*6*), surveillance of bat betacoronaviruses is crucial for understanding and assessing the spillover potential of betacoronaviruses in humans.

Bats belonging to the genus *Rhinolophus* are considered natural reservoirs of sarbecoviruses because most have been detected in *Rhinolophus* bats in countries in Asia (*3*–*8*), as well as in countries in Europe and Africa (*9*,*10*). We previously identified a bat sarbecovirus, Rc-o319, from *Rhinolophus cornutus* bats in the Iwate Prefecture of Japan, which was shown to phylogenetically belong to the SARS-CoV-2 lineage (*7*).

Vesicular stomatitis virus‒based pseudotyped virus having the Rc-o319 spike (S) protein was able to infect cells expressing *R. cornutus* angiotensin-converting enzyme 2 (RcACE2), but not those expressing human angiotensin-converting enzyme 2 (hACE2), suggesting that the Rc-o319 virus uses RcACE2 as its receptor (*7*). Sarbecoviruses detected in China and other countries in Asia were shown to vary genetically; however, the distribution and genetic variation of bat sarbecoviruses in Japan have not yet been determined.

Despite surveillance-based genetic detection of numerous bat sarbecoviruses, cultivable viruses have been rarely isolated to date, leading to the application of a pseudovirus system as described above to analyze their entry mechanisms into cells. Receptor selectivity assessed in this system does not necessarily correspond to functional receptor specificity of intact bat sarbecovirus (*11*), emphasizing the need for cultivable virus for assessment of its spillover potential of bat sarbecoviruses. We report detection, isolation, and genetic and biologic characterization of cultivable bat sarbecoviruses from several locations in Japan.

## The Study

We collected fecal samples from bats belonging to the *R. cornutus* and *R. ferrumequinum* species in Niigata, Chiba, and Shizuoka Prefectures (Appendix Figure 1, panel A). Using real-time reverse transcription PCR, we successfully detected the envelope gene sequence of sarbecovirus in 1 or 2 *R. cornutus* bat samples in each prefecture (Table 1). In contrast, all *R. ferremuquinum* bat samples were negative. These data suggested that bat sarbecoviruses are distributed among *R. cornutus* bats at various locations in Japan.

**Table 1:** Detection of sarbecoviruses in *Rhinolophus* bats by RT-PCR, Japan*

Location	Bat species	No. samples	No. positive RT-PCR samples
Niigata	*R. cornutus*	26	2
	*R. ferrumequinum*	1	0
Chiba	*R. cornutus*	11	1
	*R. ferrumequinum*	16	0
Shizuoka	*R. cornutus*	21	2
	*R. ferrumequinum*	13	0

*RT-PCR was performed by using sarbecovirus consensus primers targeting the envelope gene. RT-PCR, reverse transcription PCR.

In our previous study, we showed that a vesicular stomatitis virus‒based pseudotyped virus possessing the S protein of Rc-o319 sarbecovirus from *R. cornutus* only infected RcACE2-expressing cells, but not hACE2-expressing or other *Rhinolophus* ACE2–expressing cells (*7*). Therefore, to isolate bat sarbecoviruses, we established RcACE2-stably expressing cells (Vero-RcACE2) based on Vero/TMPRSS2 cells. Using Vero-RcACE2 cells, we successfully isolated bat sarbecoviruses, which exhibited extensive cytopathic effect with syncytium formation (Appendix Figure 1, panel B) from real-time reverse transcription PCR‒positive fecal samples from each prefecture. We designated the Niigata isolate as Rc-os20, the Chiba isolate as Rc-mk2, and the Shizuoka isolate as Rc-kw8. We further isolated the cultivable Rc-o319 strain by using Vero-RcACE2 cells.

We determined the full-genome sequence of all isolates by using next-generation sequencing and deposited the sequences in GenBank (accession nos. LC663958, LC663959, and LC663793). We found that sequence homologies were high (range 94.8%–96.8%) among all isolates from Japan (Table 2), However, Rc-mk2 and Rc-os20 lacked the entire open reading frame 8 coding region.

**Table 2:** Full-genome nucleotide identity for sarbecovirus isolates from bats, Japan

Isolate	Rc-o319	Rc-os20	Rc-kw8	Rc-mk2
Rc-o319	‒	95.6%	96.8%	94.8%
Rc-os20	‒	‒	95.4%	95.4%
Rc-kw8	‒	‒	‒	95.1%
SARS-CoV-2	81.5%	80.7%	81.4%	80.7%

**Figure 1:**
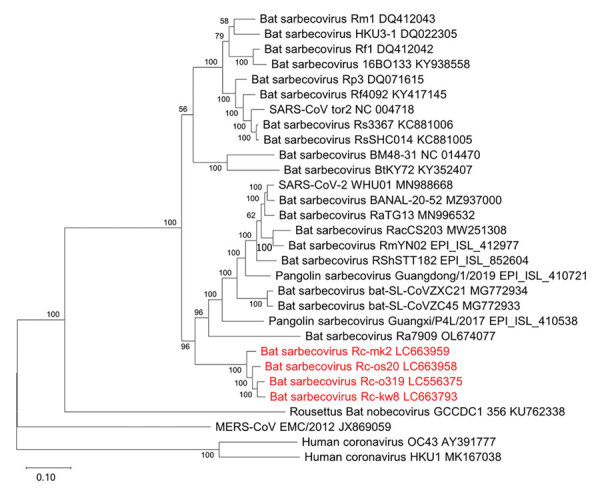
Phylogenetic tree of sarbecoviruses from bats in Japan, generated by using the full-genome nucleotide sequences with the maximum-likelihood analysis combined with 500 bootstrap replicates. Red indicates strains isolated in this study. Bootstrap values are shown above and to the left of the major nodes. GenBank accession numbers are indicated. Scale bars indicate nucleotide substitutions per site.

We also performed similarity plot analysis of entire genome sequence by using each isolate as a query, which indicated that similarities among isolates were high throughout the entire genome sequence, except for coding regions of the N-terminal domain (NTD) and receptor-binding domain (RBD) of the S gene, although NTDs of Rc-o319 and Rc-kw8 were conserved (Appendix Figure 2). No clear recombination among the isolates were observed as analyzed by RDP5 software (*12*). Phylogenetic analysis showed that the isolates from Japan formed a single genetic cluster and positioned in a clade containing SARS-CoV-2‒related sarbecoviruses, which might be designated the Japanese clade of bat sarbecoviruses (Figure 1).

We aligned the receptor-binding motif of the S protein of isolates from Japan with that of other sarbecoviruses (Appendix Figure 3, panel A). We observed that all isolates had a 9-aa deletion in this motif, as previously observed in Rc-o319, and had relatively conserved residues with Rc-o319. In addition, phylogenetic tree analysis of RBD showed that strains from Japan were included in the clade of viruses that use ACE2 orthologs as a strain receptor (Appendix Figure 3, panel B). Therefore, we assumed that these new strains from Japan use RcACE2 as a receptor.

**Figure 2:**
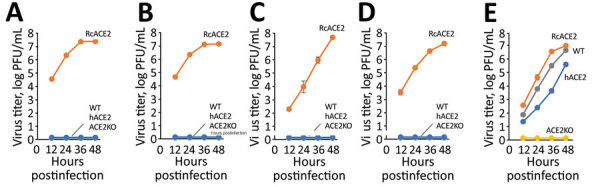
Growth kinetics of sarbecovirus isolates from bats in Japan. *Rhinolophus cornutus* bat isolates Rc-o319 (A), Rc-os20 (B), Rc-mk2 (C), and Rc-kw8 (D) or SARS-CoV-2 (B.1.1.7) € were inoculated into Vero/TMPRSS2 (WT), Vero-RcACE2 (RcACE2), Vero-hACE2 (hACE2), or Vero-ACE2KO (ACE2KO) cells at a multiplicity of infection of 0.01. The culture supernatants were collected at the indicated time points, and viral titers were determined by using a plaque assay. Data are reported as the mean titer with standard deviations from 3 independent experiments. ACE2, angiotensin converting enzyme 2; hACE2, human ACE2; RcACE2, *R. cornutus* ACE2; WT, wild-type.

To test this hypothesis, we compared the replication of isolates from Japan with that of a control SARS-CoV-2 (B.1.1.7, Alpha variant) in Vero-RcACE2, Vero-hACE2, Vero-ACE2KO, and Vero/TMPRSS2 cells. Whereas the 4 bat isolates replicated well in Vero-RcACE2 only, they did not replicate in Vero/TMPRSS2, Vero-hACE2, or Vero-ACE2KO cells, suggesting their RcACE2-dependent infectivity. In contrast, we observed that SARS-CoV-2 replicated efficiently in Vero/TMPRSS2, Vero-RcACE2, and Vero-hACE2 cells, but not in Vero-ACE2KO cells (Figure 2), suggesting multiple ACE2-dependent infectivity, including that of *R. cornutus* bats. These data suggested that at isolates from Japan use only bRcACE2 as a receptor, showing narrow host specificity.

## Conclusions

We isolated bat sarbecoviruses from *R. cornutus* bats in several locations in Japan that were phylogenetically positioned in the same cluster of the SARS-CoV-2–related viruses. These isolates used only bat ACE2 as a receptor and did not replicate in hACE2-expressing cells, forming a unique type, and suggesting a low potential for human infection.

To our knowledge, this type of bat sarbecoviruses has not been previously isolated (*13*) because African green monkey Vero cells having highly similar ACE2 to hACE2 were used for viral isolation attempts in the previous studies (*4*,*5*). Cultivable bat sarbecoviruses provide a useful and powerful tool to determine their characteristics, such as receptor specificity and pathogenicity in animals, leading to elucidation of spillover potential.

*Rhinolophus* spp. bats are relatively short-distance migrants (*14*) and lack frequent cross-contact between bat groups, explaining why most genome sequences were highly conserved among strains from Japan. Exceptions were the RBD-coding and NTD-coding regions of the S gene, which show high variation caused by immune pressure (*15*), suggesting that they diverged relatively recently from the undefined ancestral virus. Because sarbecoviruses might mutate and infect humans by intermediate hosts in wildlife or livestock, epidemiologic studies of sarbecoviruses in wildlife, including bats, need to be conducted on a long-term basis for risk assessment of their zoonotic potential.

Appendix Additional information on isolation of bat sarbecoviruses, Japan.
