# Supplementary material for: Isolation of Bat Sarbecoviruses, Japan
Source: Emerg Infect Dis. 2022 Dec;28(12):2500–3. doi: 10.3201/eid2812.220801 (PMC9707576; doi:10.3201/eid2812.220801)
Supplement: Appendix — Additional information on isolation of bat sarbecoviruses, Japan. [file 22-0801-Techapp-s1.pdf]

Article DOI: https://doi.org/10.3201/eid2812.220801

Isolation of Bat Sarbecoviruses, Japan

Appendix

Materials and Methods

Cells and Virus

Vero/TMPRSS2 cells (*1*,*2*) were kindly provided by Dr. Makoto Takeda (National Institute of Infectious Diseases, Tokyo, Japan) and maintained in Dulbecco modified Eagles medium (DMEM; Nacalai Tesque, https://www.nacalai.co.jp) supplemented with 10% fetal bovine serum, 100 units/mL of penicillin, and 100 µg/mL of streptomycin. Cells were cultured in 5% CO_2 at 37°C. SARS-CoV-2 (B.1.1.7, α variant, UT-HP127–1Nf/Human/2021/Tokyo) was propagated in Vero/TMPRSS2 cells, and aliquots were stored at −80°C.

Sample Collection

We collected 88 fresh fecal samples, 58 from *Rhinolophus cornutus* and 30 from *Rhinolophus ferrumequinum* bats living in caves, abandoned mines, or abandoned tunnels in Niigata, Chiba, and Shizuoka prefectures in Japan (Table 1). When bats were densely packed during the daytime roost, plastic sheets were placed under the roost for 1–2 h. Fresh feces that dropped onto the sheets were collected. When bats were sporadically placed during the daytime roost, we captured bats after obtaining permission from the prefectural

local governments (no. 962 for Chiba and no. 311 for Shizuoka) and kept each bat in a separate nonwoven fabric bag. Feces excreted by the bat in the bag were collected and bats were released. Fecal samples were transferred into tubes containing phosphate-buffered saline supplemented with 200 U/mL penicillin, 200 μg/mL streptomycin, and 0.25 μg/mL amphotericin B, and immediately frozen in dry ice.

Reverse Transcription PCR

RNA was extracted from the fecal samples by using the RNeasy PowerMicrobiome Kit (QIAGEN, https://www.qiagen.com), and the partial envelope gene of sarbecovirus was detected in RNA samples by real-time reverse transcription-PCR (rRT-PCR) using the RNA-direct SYBR Green Realtime PCR Master Mix (Toyobo, https://www.toyobo-global.com) and a pair of primers (5′-TCGGAAGAGACAGGTACGTT-3′ and 5′-TCGAAGCGCAGTAAGGATGG-3′) that were designed to target a highly conserved region of the sarbecovirus envelope gene.

Establishment of ACE2 Stably Expressing Cells

We constructed a plasmid, pCAGGS-blast, by inserting the XhoI-EcoRV fragment of pMXs-IRES-Bsd (*3*), which contains the encephalomyocarditis virus internal ribosomal entry site and blasticidin-resistant gene, into the *Xho*I and *Stu*I sites of the pCAGGS-MCS vector. Open reading frame sequences of RcACE2 or hACE2 were PCR-amplified from pCAGGS-RcACE2- or pCAGGS-hACE2-expressing plasmids (*4*) and cloned into *Eco*RI- and *Xho*I-digested pCAGGS-blast plasmids by using NEBuilder (New England Biolabs, https://www.neb.com). Vero/TMPRSS2 cells were transfected with pCAGGS-blast-

RcACE2 or pCAGGS-blast-hACE2 plasmids by using the PEI MAX Transfection Reagent (Polysciences, https://www.polysciences.com).

Transfected cells were treated with 10 μg/mL blasticidin S (Kaken Pharmaceutical, https://www.kaken.co.jp) 1 d posttransfection, and blasticidin S-resistant cells were selected and cloned. Highly susceptible cell clones for the pseudotyped viral infection were selected by screening using GFP-expressing VSV-pseudotyped virus possessing the S protein of Rc-o319 or SARS-CoV-2 (*4*), generating RcACE2- or hACE2-stably expressing Vero/TMPRSS2 cells (namely Vero-RcACE2 or Vero-hACE2, respectively).

Establishment of ACE2-Knockout Cells

We generated ACE2-knockout Vero/TMPRSS2 cells (Vero-ACE2KO) by knocking out the corresponding genes using the CRISPR/Cas9 system. The target sequence for the ACE2 gene (5′-TGCTGCTCAGTCCACCATTG-3′) was designed by using CRISPR direct (https://crispr.dbcls.jp) and cloned into plentiCRISPR plasmids (*5*). Addgene plasmid #52961, a gift from Dr. Feng Zhang) using NEBuilder (New England Biolabs). Vero/TMPRSS2 cells were transfected with an ACE2-targeting plasmid by using PEI MAX (Polysciences). At 24-h posttransfection, the cell supernatant was replaced with medium containing 10 μg/mL puromycin. Drug-resistant clones were randomly selected, and their genomic DNA was sequenced. Cells having insertions or deletions (in/dels) in the targeted gene were chosen for further analysis.

Isolation of Bat Sarbecoviruses

Fecal samples positive for the partial envelope gene of sarbecovirus were homogenized in TissueLyser II (QIAGEN) by using 0.1-mm glass beads (Tomy Seiko, https://bio-tomys-co-jp) in phosphate-buffered saline containing 200 U/mL penicillin, 200 µg/mL streptomycin, and 0.25 µg/mL amphotericin B. The supernatants were collected after centrifugation at 5000 × g for 5 min at 4°C and diluted 100-fold in cell maintenance medium (DMEM supplemented with 1% fetal bovine serum, 200 U/mL penicillin, 200 µg/mL streptomycin, and 0.25 µg/mL amphotericin B). Diluents were inoculated into 6-well plates containing Vero-RcACE2 cells, and plates were incubated for 60 min at 37°C after removing the inoculum. Wells were then washed once with cell maintenance medium, another 2 mL of cell maintenance medium was added to each well, and incubated at 37°C. Supernatants from cells that exhibited cytopathic effects were collected at 3–4 d postinoculation and were passed through a 0.22-µm filter. The successful isolation of viruses was confirmed by rRT-PCR, and isolates were propagated in Vero -RcACE2 cells, and aliquots were stored at −80°C.

Next-Generation Sequencing

A cDNA library was prepared from RNA extracted from bat sarbecoviral isolates by using the TruSeq Stranded Total RNA LT Sample Prep Kit Gold (Illumina, https://www.illumina.com) for Rc-mk2 and Rc-kw8 strains or the MGIEasy RNA Directional Library Prep kit (MGI, https://en.mgi-tech.com) for the Rc-os20 strain. Libraries of Rc-mk2 and Rc-kw8 strains were sequenced by using a Novaseq 6000 Sequencer (Illumina), and those of Rc-os20 were sequenced by using a DNBSEQ-G400RS

sequencer (MGI). Read sequences were mapped to the Rc-o319 genome sequence (GenBank accession no. LC556375), and sarbecoviral sequences were determined by using CLC Genomic Workbench Version 8.0.1 software (QIAGEN). Sequences of Rc-os20, Rc-mk2, and Rc-kw8 have been deposited in GenBank (accession nos. LC663958, LC663959, and LC663793, respectively).

Phylogenetic Analysis

The nucleotide sequences of sarbecoviruses were aligned by using ClustalW version 2.1 (Clustal, https://www.clustal.org). Phylogenetic trees were then constructed by performing a maximum-likelihood analysis by using EGA version X (*6*) in combination with 500 bootstrap replicates.

Evaluation of Viral Growth in Cells

Cells were inoculated with viruses at a multiplicity of infection of 0.01 and incubated for 1 h for viral adsorption. After removing the inocula, cells were incubated in cell maintenance medium, and the supernatants were collected at 12-h intervals. Viral titers were measured by using a plaque assay, in which cells inoculated with diluted viruses were overlaid and incubated with DMEM containing 1% agarose and 1% fetal calf serum for 2 d, followed by staining with crystal violet before counting plaques.

References

1. Shirogane Y, Takeda M, Iwasaki M, Ishiguro N, Takeuchi H, Nakatsu Y, et al. Efficient multiplication of human metapneumovirus in Vero cells expressing the transmembrane

serine protease TMPRSS2. J Virol. 2008;82:8942–6. PubMed https://doi.org/10.1128/JVI.00676-08

2. Matsuyama S, Nao N, Shirato K, Kawase M, Saito S, Takayama I, et al. Enhanced isolation of SARS-CoV-2 by TMPRSS2-expressing cells. Proc Natl Acad Sci U S A. 2020;117:7001–3. PubMed https://doi.org/10.1073/pnas.2002589117

3. Kitamura T, Koshino Y, Shibata F, Oki T, Nakajima H, Nosaka T, et al. Retrovirus-mediated gene transfer and expression cloning: powerful tools in functional genomics. Exp Hematol. 2003;31:1007–14. PubMed https://doi.org/10.1016/S0301-472X(03)00260-1

4. Murakami S, Kitamura T, Suzuki J, Sato R, Aoi T, Fujii M, et al. Detection and Characterization of bat sarbecovirus phylogenetically related to SARS-CoV-2, Japan. Emerg Infect Dis. 2020;26:3025–9. PubMed https://doi.org/10.3201/eid2612.203386

5. Sanjana NE, Shalem O, Zhang F. Improved vectors and genome-wide libraries for CRISPR screening. Nat Methods. 2014;11:783–4. PubMed https://doi.org/10.1038/nmeth.3047

6. Kumar S, Stecher G, Li M, Knyaz C, Tamura K. MEGA X: Molecular evolutionary genetics analysis across computing platforms. Mol Biol Evol. 2018;35:1547–9. PubMed https://doi.org/10.1093/molbev/msy096

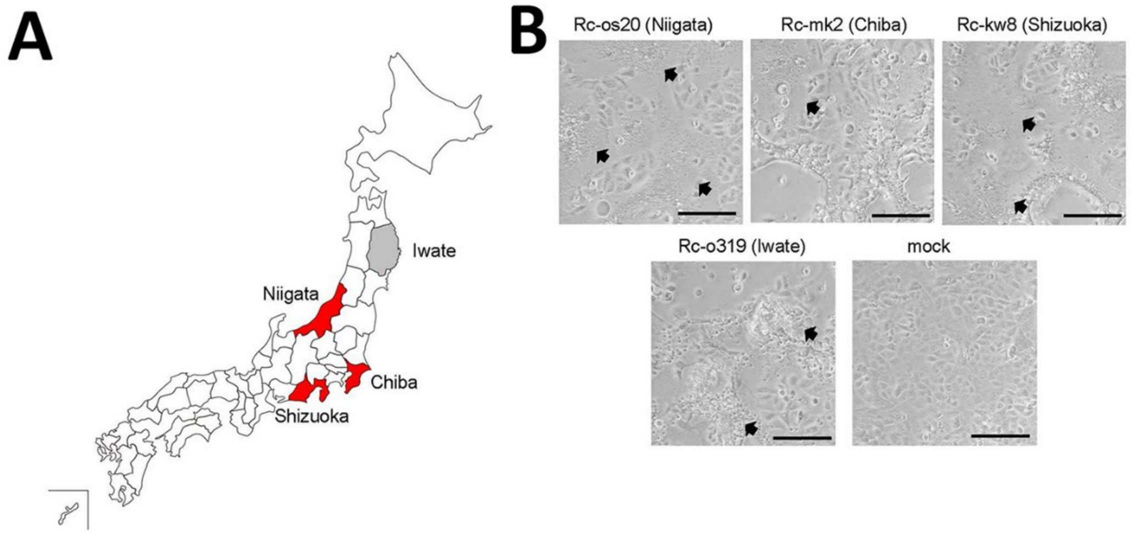

Appendix Figure 1. A) Sampling locations in Japan. Prefectures, where bats were captured, are indicated in red. Prefectures, where bat sarbecoviruses were detected in our previous study, are indicated in gray. B) Images of bat sarbecovirus-inoculated cells. Vero-RcACE2 cells were inoculated with fecal samples from *Rhinolophus cornutus* bats from several prefectures of Japan. After 1–2 d of inoculation, cytopathic effects with extensive syncytium formation (arrowheads) were observed. Scale bar indicates 200 μm.

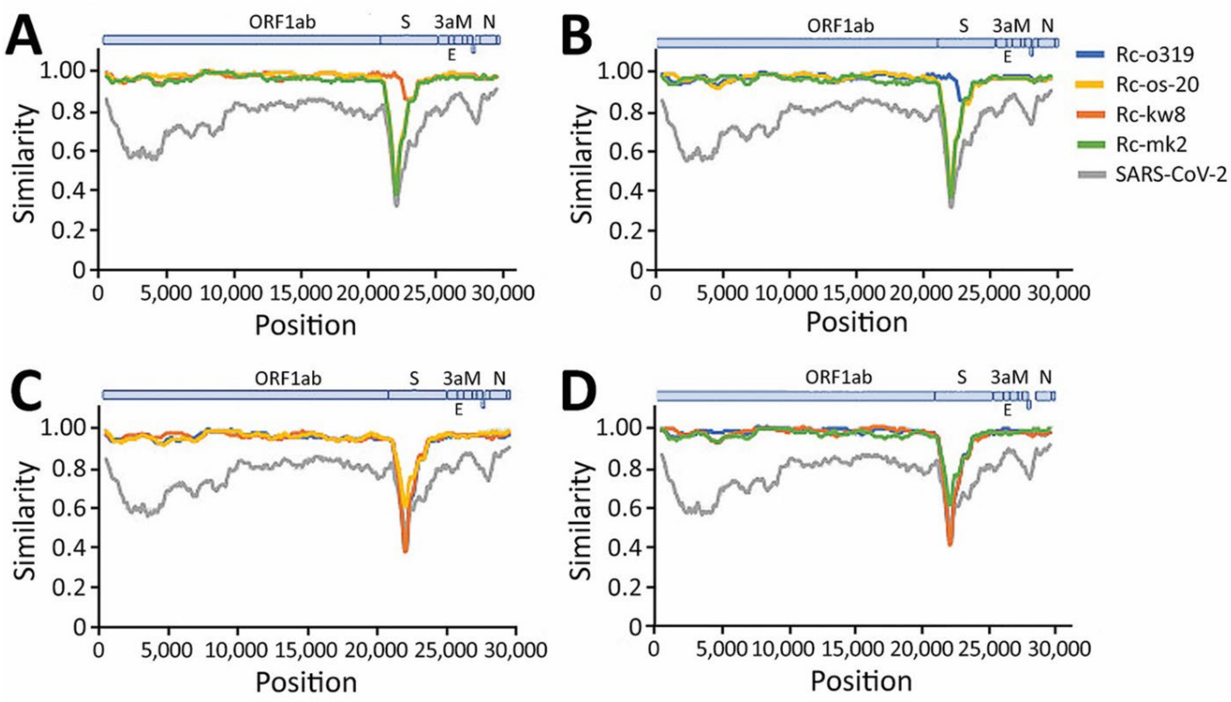

Appendix Figure 2. Genetic analysis of 4 bat sarbecovirus isolates, Japan. Similarity plot analysis of isolates was performed by using the full-length genome sequence of A) Rc-o319, B) Rc-os20, C) Rc-kw8, or D) Rc-mk2 for comparison. SARS-CoV-2 virus was used as a reference. E, envelope; M, matrix; N, nucleocapsid; ORF, open reading frame; S, spike; SARS-CoV-2, severe acute respiratory syndrome coronavirus 2.

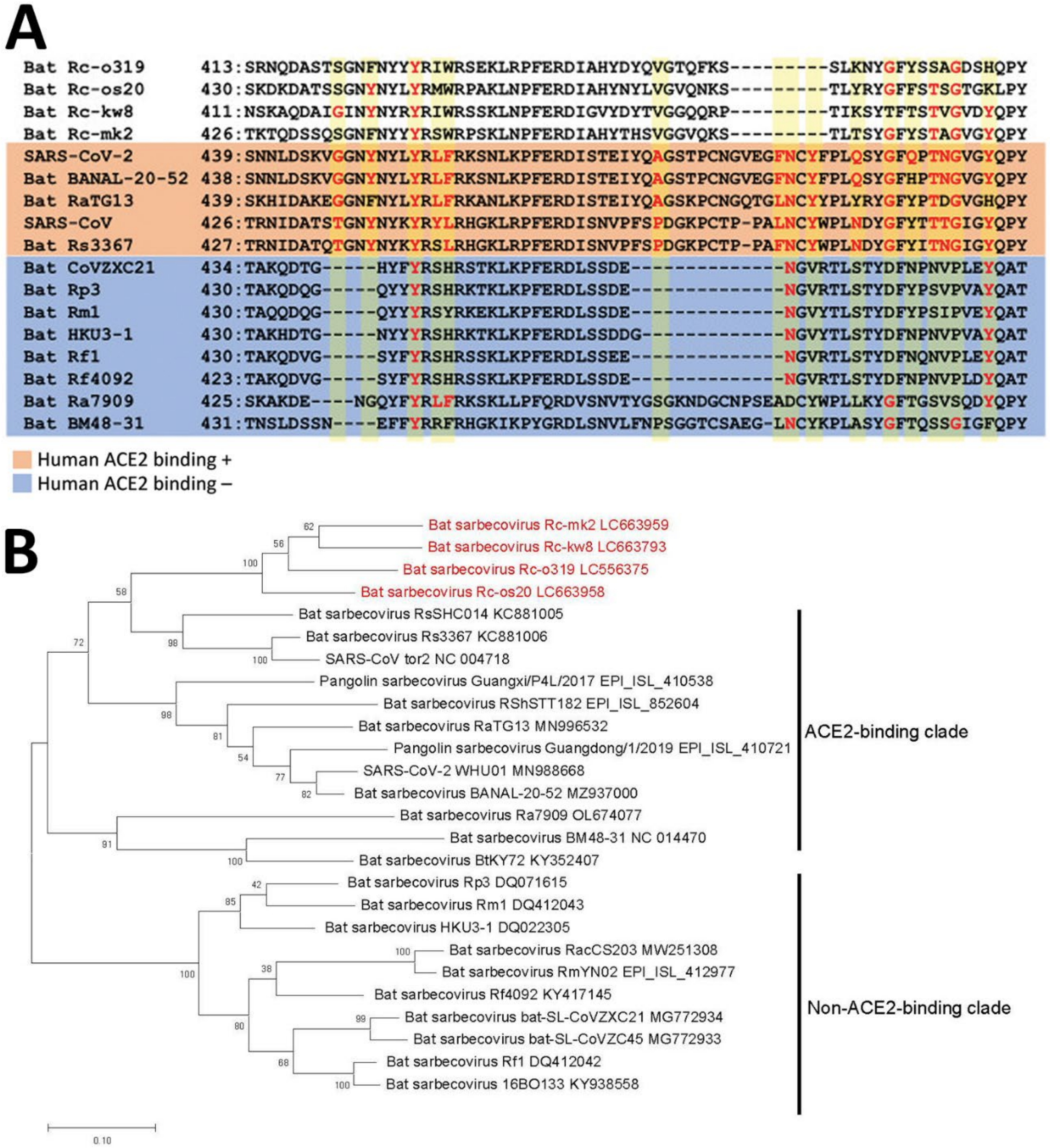

Appendix Figure 3. A) Alignment of the receptor-binding motif sequence of spike proteins of bat sarbecovirus isolates. Amino acid positions of receptor-binding motifs contacting human angiotensin converting enzyme-2 (ACE2) identified in severe acute respiratory syndrome coronavirus 2 (SARS-CoV-2) are indicated in yellow, residues identical to SARS-CoV or SARS-

CoV-2 are indicated in red, strains that are capable of binding to human ACE2 are indicated in orange, and strains that are incapable of binding to human ACE2 are indicated in blue. B) Phylogenetic tree of bat sarbecoviruses was generated using the receptor binding domain (RBD) nucleotide sequences with the maximum-likelihood analysis combined with 500 bootstrap replicates. Red text indicates the isolates in this study. Bootstrap values are shown above and to the left of the major nodes. Scale bars indicate nucleotide substitutions per site.
